# Indigenous mental health therapies

**DOI:** 10.1192/j.eurpsy.2021.862

**Published:** 2021-08-13

**Authors:** L. Mehl-Madrona, B. Mainguy

**Affiliations:** 1 Medical Arts And Humanities Program, University of Maine, Orono, United States of America; 2 Education Division, Coyote Institute - Canada, Ottawa, Canada

**Keywords:** Indigenous people, psychotherapy, Four Directions, Culture

## Abstract

**Introduction:**

Cultural differences exist among indigenous and mainstream peoples about the nature of mind and how one achieves mental health.

**Objectives:**

We aimed to determine what is important and different for indigenous communities from non-indigenous communities.

**Methods:**

We assembled a focus group of 109 indigenous and non-indigenous mental health counselors who worked in indigenous communities to meet weekly for 90 minutes via an internet platform (Zoom) for 810weeks with asynchronous communication between meetings.

**Results:**

The metaphor of the Four Directions, represented with different colors, attributes, and animals, was important in indigenous communities. Participants emphasized the idea of relational, non-local mind which places identity in the relationships between people rather than an individual body. Illnesses were seen as conscious beings who visit people and bring teachings. The healing, participants said, comes from reaching within the suffering and the pain to find the answer from within which makes meaning from an illness. People are expected to make offerings and sacrifices to the spirit of the illness to move toward wellness. These sacrifices can include lifestyle changes that the person might otherwise not make. Using substances without the proper protocols and prayers was likened to sorcery or witchcraft which can become a powerful incentive to stop disrespecting these substances and to find meaning in setting them aside with the help of a supportive community.

**Conclusions:**

What participants saw as important for indigenous populations was different from what is usual for non-indigenous mental health services. Participants stressed the importance of non-indigenous providers understanding this and not dismissing these ideas.

